# Mental Health Services in Rural China: A Qualitative Study of Primary Health Care Providers

**DOI:** 10.1155/2015/151053

**Published:** 2015-12-24

**Authors:** Zhenyu Ma, Hui Huang, Qiang Chen, Faqin Chen, Abu S. Abdullah, Guanghui Nie, Qiming Feng, Bo Wei

**Affiliations:** ^1^School of Public Health, Guangxi Medical University, Nanning, Guangxi 530021, China; ^2^Guangxi Zhuang Autonomous Region Brain Hospital, Liuzhou, Guangxi 545005, China; ^3^Youjiang Medical University for Nationalities, Baise, Guangxi 533099, China; ^4^Boston University School of Medicine, Boston Medical Center, Boston, MA 02118, USA

## Abstract

This study aimed to understand the challenges that primary health care providers faced in the process of delivering mental healthcare and assess their attitudes towards patients with mental health problems. In-depth interviews were conducted among 42 primary health care providers in two counties of Guangxi province, China. All interviews were audio-recorded and analyzed thematically. Primary health care providers in both counties faced the same difficulties: lack of professional knowledge, fear of patients' attack, more extra work, and less subsidies. However, most of primary health care providers (30/42) were still willing to do mental healthcare management. All the interviewees considered that communication skills with patients and their family members, proper attitude (without discrimination), and the professional knowledge of mental health are required. There are still several participants (15/42) who showed negative attitude toward mental disorders. Nearly all the respondents (39/42) emphasized the importance of increasing their income or subsidies by the government. This qualitative study provides insights into mental health services in rural communities of Guangxi and identified issues that could be considered in engaging primary health care providers in the management of mental disorders.

## 1. Introduction

Mental health conditions affect millions of people in the world. The World Health Organization (WHO) estimates that 151 million people suffer from depression and 26 million people from schizophrenia; 125 million people are affected by alcohol use disorders [1]. In low-income countries, depression represents almost as large of a problem as does malaria (3.2% versus 4.0% of the total disease burden) [2]. The WHO advocates that mental health services should be available in the grassroots level and integrated systematically into primary health care (PHC) [3]. Community-based services are widely regarded as the best approach for providing mental health treatment and care. At the same time primary health care providers are the basic health service resources, as well as the only resource of mental health service in rural areas. The Chinese Mental Health Work Plan (2012–2015) was outlined to equip 90% of community health service centers or conditional township health centers with full-time or part-time medical personnel who would engage in delivering care for mental health [4].

Guangxi is a less developed province in China and lacks mental health professional, especially in rural communities. At the same time, the available mental health services that are available in the city level hospital, mainly through the psychiatric departments, are not reachable by the rural population due to financial hardships, stigma, and distance [5]. Our earlier study reported a high prevalence of mental disorders (26.81‰) and less utilization of health care services or self-medication among patients living in rural Guangxi [6]. Thus, the improvement and development of community mental health care has to rely on the primary health care providers, also known as “village doctors” who are usually from the same community and are familiar with the community members in the area. Primary health care providers not only are the general practitioners of primary health care but also play a mediating role among psychiatric institutions, psychiatrists, and the community population. Without this medium, psychiatrists are difficult to carry out mental health service at the grassroots level. As one of the indispensable groups in primary health care, primary health care providers' attitude towards the mental disorder patients would greatly affect the quality of health service. Evidence based guidelines for nonspecialists to enable them to identify and manage mental disorders were being carried out in low- and middle-income countries [7]. However, there were lack of understanding of the challenges that primary health care providers faced in the process of carrying out mental health work and their attitudes towards mental disorders. Thus, we conducted this qualitative study to compare the primary health care providers between two different mental health service models in rural Guangxi by conducting in-depth interviews (IDIs). The study aims to understand the challenges that primary health care providers faced in the process of carrying out mental health work and to realize their attitudes towards mental patients and so forth. This study is of great importance to guide the community mental health services in rural China.

## 2. Methods

### 2.1. Sample and Settings

There were two models of mental health services in rural Guangxi: one is hospital-community integrated service model and the other is the psychiatric hospital-centric model. We selected participants from the two model areas. In this study, the sample area is Liujiang County, the proportion of the population accounted for 89.7% of Zhuang ethnicity. The National Continuing Management and Intervention Program for Psychoses (686 Program) has been developed in Liujiang County since 2006 [8]. With a new hospital-community integrated service model, this program has actually covered 500000 general population in 11 towns. However, Liucheng County is the control area in this study, and its socioeconomic development level and capacity of rural health services are comparable with Liujiang County, and it still follows the traditional hospital-centric model of community mental health services.

Participants were primary health care providers engaged in community mental health services who were randomly selected from Liujiang and Liucheng Counties, respectively.

### 2.2. Procedures

Participants were recruited, during June-July 2013, through the hospital liaisons in each township health center. Interviewers introduced themselves as being researchers from the School of Public Health of Guangxi Medical University conducting a study on primary mental health services. Researchers then explained the format of the in-depth interviews (IDIs) and provided assurance that all responses would be completely anonymous. The IDIs were conducted in a location convenient for the participants and took about 60 minutes.

### 2.3. Data Collection

A team of researchers with experience in conducting qualitative research collected the data. All of the IDIs were conducted in Mandarin Chinese or the local Guangxi dialect using a semistructured interview guide and audio-recorded. Open-ended questions were used in a semistructured interview approach. The guide included questions and queries on the following six themes: the situation of mental health service in PHC, main difficulties to provide mental health care, willingness to provide mental health care, required abilities of the primary health care providers, attitude toward mental disorders, and how to improve mental health care in rural area. Interviewers were graduates students at the School of Public Health of Guangxi Medical University and attended a 3-day training course on qualitative research methods and rural mental health services research. The training also included a session on the ethical aspects of human subject research. All the participants provided their written informed consent, including guidelines, relevant risks, and benefits. To collect data, interviewers recorded the session with a digital voice recorder under permission from the participants. To compensate for their time, each participant was given a cash amount of RMB 50 (US$8). The study was approved by the Ethics Committee of the Guangxi Medical University.

### 2.4. Analysis

The interviewers discussed and summarized the content of each IDI and reviewed the notes taken immediately after the IDI. These debriefings were useful (i) to identify most important themes and ideas and (ii) to assess the need for any modification in the subsequent IDIs. Audio recordings of each interview were transcribed verbatim into Chinese. Content analysis was conducted following the guidelines outlined in Qualitative Interviewing [9]. Two members of the research team coded each transcript independently, with discrepancies resolved through consensus. The process of coding involved identifying key themes and marking these out on the transcripts [10]. All additional notes taken during the course of the IDIs were examined to identify various themes presented in these qualitative discussions. Frequencies were used only in a general sense such as most, some, or a few.

## 3. Results

A total of 42 IDIs were conducted among primary health care providers from Liujiang and Liucheng counties. 31 (73.8%) of the participants were males and 11 (26.1%) were females. Education varied from high school or below (52.4%) to junior college or above (47.6%). The average length of working time was 21 years (Table 1). There was no statistical difference (*P* > 0.05) between the two groups in marriage, income, gender, educational level, and working time.

The findings revealed six main themes relating to mental health services in rural communities: the situation of mental health service in PHC, main difficulties to provide mental health care, willingness to provide mental health care, required abilities of the primary health care providers, attitude toward mental disorders, and how to improve mental health care in rural area. These themes are described below supplemented by participants' statements on key themes provided in Table 2.

### 3.1. The Situation of Mental Health Service in PHC

There were different working models of mental health services in the two counties. Liujiang had implemented a new hospital-community integrated service model with the support of National Continuing Management and Intervention Program for Psychoses (called “686 Program”) since 2006. Most of the primary health care providers in Liujiang were trained for managing severe mental illnesses. They were more knowledgeable and familiar with the management and preventive services including community education, observation, and follow-up. However, Liucheng County was still under the traditional hospital-centric model of community mental health services. The management of severe mental illnesses in Liucheng was still relatively lagging behind.

For example, one interviewee in Liujiang said, “my main job is to follow-up the mental disorders, observe their medication compliance and their life status. If they have drug side effects or abnormal conditions, I will contact with superior hospital. As the administrator of severe mental illnesses, I play a role of bridge and tie between hopital and mental ill patients, such as telling the patients to take physical examination, and to take a follow-up visit at any time. If there is something abnormal, I need to report to the superiors in time. In addition, I also should concern about the mental ill patients, talk to them and their family members with the life and job topics, in order to ease their tensions.” Another said “I had a patient. He comes to the primary health center every month. I give the same medications that the psychiatrist used to give. He has been well for five years now, with no need to return to the psychiatric hospital.”

But one participant in Liucheng said, “generally, I do what the township health center arranged. To telling the truth, I am rarely active to do the work, because I have many other things to do, such as managing the diabetes and hypertensions. Another reason is that there is no attack by mental illnesses in my village. So I don't need to care about them.” Another PHC provider said, “formerly, the management of the mental illnesses was almost nothing. Last year I attended a meeting that I was asked to look for the mental illnesses and fill up the health forms. But we have had no management approach yet.”

### 3.2. The Main Difficulties to Provide Mental Health Care

PHC providers both in Liujiang and Liucheng face the same difficulties in the process of providing mental health service. They said that the lack of professional knowledge, fear of being attacked by patients, more extra work, and less subsidies made mental health care service difficult.

Some of participants (15/42) described mental health care service as a difficult task. One of them said, “I do want to help patients by providing mental health care, but what can I do for them without any basic knowledge of psychiatry.”

Fear of mental disorders affected PHC providers (especially the females) in providing mental health care. A female participant said, “I am afraid of the mental disorders experience relapses. The patients could not well understand our work, sometimes they curse and shout us.”

More than half of participants (28/42) considered that the subsidies were too low to reflect the job's value. “The job had no subsidies in the past two years. However, we had to do the work without subsidies.” Another voice was, “it takes 50 minutes by motorcycle to the far most village. How to reimburse the visiting fare is also a problem.”

### 3.3. Willingness to Provide Mental Health Care

Even though there were kinds of difficulties during mental health care service, most of PHC providers (30/42) were still willing to do it in both counties. They considered mental health care as their duty and responsibility. One said that “it is my responsibility to do it. When I saw some of mental illnesses could go back to farm work after taking pills, I have a sense of achievement.” Other participants (8/42) thought they have to provide mental health care by the upper-level administrative requirement. Only few respondents (4/42) showed that they were not willing to provide mental health care, like saying “I don't want to do it. Nobody will work without pay. All the work that I did has no value at all.”

### 3.4. Required Abilities of the Primary Health Care Providers

All the interviewees considered that the most important factor of mental health service in rural area is communication skills with patients and their family members. Then the appropriate attitude (without discrimination) and the professional knowledge of mental health are also required. One experienced PHC provider in Liujiang said, “the ability to communicate should be skillful. Firstly you should greet him. Never talk with him about the illness immediately. Talk about other things before settling down to discuss the current illness. As you are friendly with the patients, they will answer your questions about illness. For example, I used to ask the patient whether he had some porridge or not (breakfast habit in rural Guangxi), he said yes. Then I asked what he had done just now, he said he had done the farm work. Next you could ask him about the crop of sugarcane. Make sure the patient is relaxed and happy, he will tell you his illness. At that time, you can ask him which kind of medicine he take, how often he take the medicine every day, and so forth. It's the same way to find new cases suffered mental disorders. When you communicate well with the villagers, they will tell you something new.”

### 3.5. Attitude toward Mental Disorders

There are still several participants (15/42) who showed negative attitude toward mental disorders during the interview. Most of them are scared of a sudden attack by patients especially the manic and schizophrenic. One said, “most of patients are normal and follow the doctor's advice. But they still may suffer a episode of mental disorder by any irritation. It is not as safe as you provide health care to the hypertension and diabetes.” Another said, “my family member worry about me to take care of mental illnesses. They worry about my safety.” An unmarried female participant even said, “I am afraid of having problems with my dating, when they know that I regularly contact with mental disorders. They won't like me to do this kind of job. People usually have discrimination against those who serve the mental health patients.”

### 3.6. How to Improve Mental Health Care in Rural Area

Participants provided suggestions to improve mental health care in rural area according to their practical experience. Increasing financial support was most common among the PHC providers. Every participant in Liujiang hoped that the “686 Program” would continue by with sustainable financial support. Nearly all the respondents (39/42) emphasized—directly or indirectly—the importance of increasing their income or subsidies by government, as one participant said, “if the government increased subsidies, it would be quite a motivation for village doctors to do mental health care in an active way.”

## 4. Discussion

PHC providers engaged in community mental health services are vital to increase the availability of mental health interventions across the rural population. This quantitative research helps us to better understand health workers' attitude and notions about providing primary mental health care. The results show that there is different working status of mental health services in two counties. The PHC providers in Liujiang County with 686 Program do much more mental health interventions, including psychosocial strategies, care management, pharmacological strategies, and reducing severity of symptoms, than that of Liucheng County. The National Continuing Management and Intervention Program for Psychoses (known as the “686 Program”) has provided the standardized trainings for village doctors in Liujiang to make them participate in the prevention and control network of the mental patients, such as involvement in mental disease management, mental health education, and social treatment and prevention of mental illness [11]. As a result, mental health service in Liujiang was better. The 686 program drives and motives the community mental health work. National Continuing Management and Intervention Program for Psychoses plays an important role in the development of rural community mental health service. Collins et al. also pointed that intergrating care for mental disorders into chronic disease care could reinform health services and reduce cost [12].

Lack of professional knowledge, fear of patients' attack, more extra work, and less subsidies were obstacles in providing mental health service. However, most of PHC providers were still willing to provide primary mental health care due to their professional responsibility. During the last two years, research on other 686 program pilot areas indicated that it should develop official in-service training on mental health by means of this program platform to improve the PHC providers' mental health knowledge and the ability of recognizing early symptoms among common mental disorders [13]. A recent World Health Organization and World Organization of Family Doctors report also identified that countries should improve health workers' knowledge, skills, and confidence to provide mental health interventions [14]. The respondents in our interview also pointed out that skills and knowledge of mental health were required as case managers. Improving their correct mental health cognition not only can reduce the mental patients' fear, segregation, and discrimination, but also can reduce severity of symptoms and improve daily functioning in work, social, and community life by effective mental health care management.

The findings show that some PHC providers had negative attitude toward mental disorders. Health care workers are the main workforce of providing professional health care, and their attitude towards the mental disorders is the key factor that influences the patients' recovery and promotes their social functional recovery. Health workers' attitudes affect the way of treating the patients, while psychiatric workers' attitudes are influenced by patients' symptoms [15, 16]. Inappropriate attitude towards mental disorders and their family members is a common and significant social problem. The primary mental health care providers should pay attention to this and should correct people's inappropriate attitudes. Although they provide mental health services for the patients, the providers' negative attitude towards the mental disease not only affects the way of working, such as passive working attitude, little patience, and reluctance, but also affects the patient's self-assessment and treatment compliance, reducing the result of treatment by the providers.

Nowadays, with the rapid development in science, technology, and civilization, many people including some PHC providers still have pessimistic and negative attitudes towards mental disorders. That is to say, it is very urgent task to popularize correct mental health knowledge and establish the mental health laws and regulations. PHC providers play an important role in connecting the villagers with health care institute. Government should increase the primary mental health care workers' income and welfare to eliminate unsatisfied factors existing in the work; at the same time, the appropriate incentives should be made, which provides trainings and reeducation opportunities and development space for the PHC providers, in order to improve their work enthusiasm.

Strengths of our study were not only the diverse range of respondents in terms of age, gender, education, and working years, but also the location (two counties with different mental health care model) to gather varying views. Also one limitation was that all participants were recruited from the less developed area Guangxi province, limiting the generalizability of the findings to attendees in other developed areas of China. However, this is the first quantitative study focusing on the thoughts and attitudes among primary mental health care providers in rural China. The findings should encourage more research in this area to promote primary health care based mental health care in rural China.

## Figures and Tables

**Table 1:** Demographic characteristics of in-depth interview (IDI) participants (*n* = 42).

Characteristics	In-depth interviews (*n* = 42)	
	Liujiang (*n* = 21)	Liucheng (*n* = 21)
Gender		
Males	16	15
Females	5	6
Age (mean ± SD)	46.8 ± 18.1	45.4 ± 15.9
Education		
High school or below	17	15
Junior college or above	4	6
Years of work (mean ± SD)	21.7 ± 13.7	19.9 ± 11.0

**Table 2:** Typical statements made by PHC providers by key themes.

	The situation of mental health service in PHC	Main difficulties to provide mental health care	Willingness to provide mental health care	Required abilities of the primary mental health providers	Attitude toward mental disorders	How to improve mental health care in rural area
Liujiang County with the hospital-community integrated service model	As the administrator of severe mental illnesses, I play a role of bridge and tie between hospital and mental ill patients, such as telling the patients to take physical examination, and to take a follow-up visit at any time.	I am afraid of the mental disorders experience relapses. The patients could not well understand our work, sometimes they curse and shout us.	As long as I have certain knowledge to help the patients, I would like to do.	On one hand, knowledge and skill of mental health, on the other hand, the abilities of diagnosis and treatment, the skills of communication, and so forth.	I treat mental patients as normal persons. I think we should mobilize people who around them to care about them.	The most important thing is that rural mental health care, as well as our village doctors, should be recognized by the society and supported by the government.
	I take the family visits of mental illnesses 2-3 times every month, as well as health education, and record the patient's progress. Any condition changes I will contact the superiors. It is important to recognize the symptoms and to nip it in the bud.	It takes 50 minutes by motorcycle to the farest [*sic*] village. I asked the family members to come to establish medical records. They did not. How to reimburse the visiting fare is also a problem.	It is my responsibility to do it. When I saw some of mental illnesses could go back to farm work after taking pills, I have a sense of achievement.	Ability of communicate should be skillful. Firstly you should friendly talk about daily life, such as breakfast, farm work, crop of sugarcane, and so forth. Never asking illness immediately. Make the patient relax and happy. Then you can ask him what medicine he takes, how often and so on.	As a village doctor, I hope the mental disorders get proper treatment, recovery, live and work independently. I am happy if I am helpful in this.	I hope government pay more attention to improve our ability by increasing income, developing training and raising satisfaction, and so forth. Village doctors' abilities are very important in mental health work.
	I am responsible to interview the mental disorders and observe their behaviors, to supervise whether they take medicine on time, and to report at any time.	The job had no subsidies in the past two years. However, we had to do the work without subsidies. No insurance, nothing.	I like to do this work not only for mental illnesses but also for people in our village.	At least PHC providers should know basic mental knowledge, and have the ability of communication. Don't stigmatize.	Actually I think we do not care enough about mental disorder people and their family members.	The treatment of mental illness costs a lot while patients living in rural have no more money. Financial support is essential.

Liucheng County with the psychiatric hospital-centric model	To telling the truth, I am rarely active to do the work, because I have many other things to do, such as managing the diabetes and hypertensions.	The family members don't cooperate with our door-to-door visits, and I am lack of professional skills. As a result, I don't know how to help them.	I don't want to do it. Nobody will work without pay. All this work was for nothing. No value.	We need to get more basic knowledge, and take trainings of mental health. We are lack of basic knowledge.	I do not discriminate the mental illnesses. They have no differences with us when they take medicine.	If the government increases subsidies, it will be quite a motivation for village doctors to do mental health care in an active way.
	Formerly, the management of the mental illnesses was almost nothing. Last year I attended a meeting that I was asked to look for the mental illnesses and fill up the health forms. But we have had no management approach yet.	The follow-up visit is required each month, but the subsidies of the extra work are so little. I have to cope with an ever-expanding workload without money.	I do love this job even it is very dangerous. It is very difficult for PHC providers to do well without governmental supports.	One point is professional ethics, the other point is mental strength and nice personality. What is more, the good communication with patients' family members is also needed, as well as updated personal knowledge and skills.	Frankly speaking, I am afraid of mental disorders' attack. Especially the manic patients. Even though it is dangerous, it is still my duty to contact with them.	Government should pay more money on the treatment of mental disorders. Free medicine for outpatients and reinbursement [*sic*] of hospital bills for severe mental illnesses should be provided.
	I do what the superiors ask me to do. Such as writing the materials and giving guidance to the family members of mental illnesses.	I don't know much about mental health knowledge. I think I need to learn, to train, and then to manage the patients.	I have to do mental health care as a PHC provider whether there is good condition or not.	PHC providers should go on well with the villagers. That is to say, we should not only communicate with them, but also know the clinical knowledge.	I do not like to contact with mental disorders. They are abnormal and maybe dangerous.	Well, we do have policy to improve mental health in rural by central government. But very little has been achieved in the implementation at grass-roots level. This aspect should be strengthened.
